# A Bayesian Model for Paired Data in Genome-Wide Association Studies with Application to Breast Cancer

**DOI:** 10.3390/e27101077

**Published:** 2025-10-18

**Authors:** Yashi Bu, Min Chen, Zhenyu Xuan, Xinlei Wang

**Affiliations:** 1 Department of Mathematical Sciences, University of Texas at Dallas, Richardson, TX 75080, USA; 2 Department of Biological Sciences, University of Texas at Dallas, Richardson, TX 75080, USA; 3 Department of Mathematics, University of Texas at Arlington, Arlington, TX 76019, USA

**Keywords:** single nucleotide polymorphism, somatic mutation, TCGA

## Abstract

Complex human diseases, including cancer, are linked to genetic factors. Genome-wide association studies (GWASs) are powerful for identifying genetic variants associated with cancer but are limited by their reliance on case–control data. We propose approaches to expanding GWAS by using tumor and paired normal tissues to investigate somatic mutations. We apply penalized maximum likelihood estimation for single-marker analysis and develop a Bayesian hierarchical model to integrate multiple markers, identifying SNP sets grouped by genes or pathways, improving detection of moderate-effect SNPs. Applied to breast cancer data from The Cancer Genome Atlas (TCGA), both single- and multiple-marker analyses identify associated genes, with multiple-marker analysis providing more consistent results with external resources. The Bayesian model significantly increases the chance of new discoveries.

## 1. Introduction

Many complex diseases, including diabetes, heart disease, hypertension, and various cancers, are associated with genetic factors. Identifying the genetic factors in complex diseases is critical to understanding disease heritability, which may lead to better strategies of the diagnosis and the treatment of disease. Single nucleotide polymorphisms (SNPs)—single-base substitutions in the genome—account for nearly 90% of genetic variation [1]. Over the past decade, genome-wide association studies (GWASs) have emerged as a powerful approach for linking common variants to complex diseases such as breast cancer, Crohn’s disease, bipolar disorder, and hypertension [2,3,4]. Standard GWASs typically adopt a case–control design, sampling controls from the healthy population and cases from the affected population, to identify disease-associated SNPs across the genome. However, these studies explain only a small proportion of heritability [5].

Paired tumor–normal data—where tumor and normal tissues from the same patient are genotyped—focus on somatic mutations driving cancer development. Resources such as The Cancer Genome Atlas (TCGA) provide extensive paired data for numerous cancer types, including breast cancer. While many mutations have no phenotypic effect, some drive cellular dysfunction and tumor development [6]. It has been reported that most cancer genes harbor somatic mutations [7,8]. Often the development of cancer is a progressive process in which multiple mutations are accumulated in a normal cell that eventually evolves to a cancerous cell, which can evade the immune mechanisms and start to proliferate. Understanding the role of somatic mutations in carcinogenesis can improve risk prediction, monitoring, early detection, and personalized therapy.

The TCGA has been collecting tumor tissues along with their matched normal samples for different types of cancer since 2005. More than 30 cancer types are involved in genomic characterization and sequence analysis in the TCGA project. Unlike traditional case–control GWASs, paired tumor–normal data inherently control for genetic background and shared environmental factors, reducing confounding and potentially improving power. Yet conventional GWAS methods assume independence between cases and controls and are not directly applicable to paired data. Additionally, single-locus GWASs often miss moderate-effect variants due to stringent multiple testing thresholds and fail to capture joint SNP effects.

In this paper, we propose a novel framework for analyzing somatic mutations using paired tumor-normal data. We first introduce a penalized maximum likelihood estimation (MLE) for single markers and then develop hypothesis tests based on the model. To improve the model performance, a hierarchical Bayesian model is developed for single-marker analysis. To further increase power to detect associated variants, we extend the hierarchical Bayesian method to multi-marker analysis to detect joint effects. Our approach enhances the detection of somatic mutation associations and joint effects to better understand the genetic risks. Finally the single- and multi-marker analysis are applied to breast cancer data using hierarchical Bayesian models.

## 2. Materials and Methods

### 2.1. Single-Marker Analysis

In a matched-pair design, each patient provides both tumor (case) and adjacent normal (control) tissues. This structure enhances the detection of somatic mutations and controls for inter-individual variability. However, the common GWAS method requires that cases and controls are independent, and thus are unsuited for the matched-pair data. We present novel approaches to developing single-marker analysis using frequentist and Bayesian methods.

At a locus, let *A* be the risk allele frequency, and *M* be the somatic mutation rate. Under genetic equilibrium, the probabilities of carrying genotype 0, 1, 2 are ${\left(1-A\right)}^{2},\;2A\left(1-A\right),\;A^{2}$, respectively. The mutation rate *M* characterizes the probability of an allele alteration at the locus. Given the normal tissue genotype is 0, the mutant genotypes can be 0, 1, and 2 with probabilities of ${\left(1-M\right)}^{2},\;2M\left(1-M\right),\;M^{2}$, respectively. If the normal genotype is 1, the mutant genotype can be 0, 1, and 2 with probabilities of $M\left(1-M\right),\;M^{2}+{\left(1-M\right)}^{2},\;M\left(1-M\right)$, respectively. If the normal genotype is 2, the mutant genotypes can be 0, 1, and 2 with probabilities of $M^{2},\;2M\left(1-M\right),\;{\left(1-M\right)}^{2}$, respectively.

Next, consider the disease risk. The penetrance is defined as the probability of having cancer given a specific genotype:

$$\begin{array}{c} \pi_{t}=P\left(Disease|Mutated\;genotype=t\right),\;t=0,\;1,\;2. \end{array}$$

If the mutant is unassociated with disease, $\pi_{2}=\pi_{1}=\pi_{0}$. If the mutant is a risk allele, $\pi_{2}\geq \pi_{1}\geq \pi_{0}$. Let $R_{t}$ be the allelic relative risk (RR) given a specific genotype: $R_{t}=\pi_{t}/\pi_{0},\;t=$1, 2. If the somatic mutation are not associated with cancer, the corresponding relative risk should be one. Here we consider the additive genetic risk model $R_{1}=\left(R+1\right)/2$, and $R_{2}=R$, where *R* is the relative risk parameter. Other genetic risk models, such as dominant, recessive, and multiplicative can be considered as well. Thus, the expected probability of paired genotypes in the patient population can be derived as follows. Let $P_{i,j}$ be the the probability of the paired normal–tumor genotype, where i=0, 1, 2 is the normal tissue genotype and j=0, 1, 2 is the tumor tissue genotype. Each $P_{i,j}$ is a function of (R,A,M), i.e., $P_{i,j}=f_{i,j}\left(R,A,M\right)$, as defined below:

(1) $$\begin{array}{cc} & P_{00}=\frac{{\left(1-A\right)}^{2}{\left(1-M\right)}^{2}}{q_{sum}},\\ \; & P_{01}=\frac{2{\left(1-A\right)}^{2}M\left(1-M\right)}{q_{sum}}\cdot \frac{R+1}{2},\\ \; & P_{02}=\frac{{\left(1-A\right)}^{2}M^{2}R}{q_{sum}},\\ \; & P_{10}=\frac{2A(1-A)M(1-M)}{q_{sum}},\\ \; & P_{11}=\frac{2A\left(1-A\right)[M^{2}+{\left(1-M\right)}^{2}]}{q_{sum}}\cdot \frac{R+1}{2},\\ \; & P_{12}=\frac{2A(1-A)M(1-M)R}{q_{sum}},\\ \; & P_{20}=\frac{A^{2}M^{2}}{q_{sum}},\\ \; & P_{21}=\frac{2A^{2}M\left(1-M\right)}{q_{sum}}\cdot \frac{R+1}{2},\\ \; & P_{22}=\frac{A^{2}{\left(1-M\right)}^{2}R}{q_{sum}}, \end{array}$$

where $q_{sum}$ is the normalization constant such that $\sum_{i}\sum_{j}P_{i,j}=1$. Note that $q_{sum}$ is also a function of *R*, *A* and *M*.

Suppose a simple random sample of size *n* is drawn from all patients. Therefore, the joint distribution of normal–tumor genotypes of a SNP follows a Multinoulli distribution, also known as the generalized Bernoulli distribution.

#### 2.1.1. Maximum Likelihood Estimation

We first consider a penalized maximum likelihood estimation for *R*, *A* and *M*. For each genetic marker, the sample data can be summarized to 9 counts, i.e., $n=(n_{00},n_{01},n_{02},n_{10},n_{11},$$n_{12},n_{20},n_{21},n_{22})$, where $n_{ij}$ is the number of patients whose normal tissue genotype is *i* and tumor tissue genotype is *j*. Assuming a simple random sample from all patients, n will follow the Multinoulli $\left(n,(P_{00},P_{01},P_{02},P_{10},P_{11},P_{12},P_{20},P_{21},P_{22})\right)$. The likelihood function is written as follows:

$$L\left(R,A,M|n\right)=\prod_{i=0,1,2}\prod_{j=0,1,2}{\left(f_{i,j}\left(R,A,M\right)\right)}^{n_{i,j}}$$

To prevent the parameter estimations on the boundary, we add a penalty term to the log-likelihood function. The boundaries are set so that R∈(0, ∞), A∈(0, 1), and M∈(0, 1). The penalized log-likelihood function is

$$\begin{array}{cc} l_{p}\left(R,A,M|n\right)= & \sum_{i=0,1,2}\sum_{j=0,1,2}n_{i,j}\;log\left(f_{i,j}\left(R,A,M\right)\right)-\lambda \;log^{2}\left(A\right)\;-\;\lambda \;log^{2}\left(1-A\right)\\ & -\lambda \;log^{2}\left(M\right)-\lambda \;log^{2}\left(1-M\right)-\alpha \;log^{2}\left(R\right), \end{array}$$

where α and λ are tuning parameters. When the estimations of parameters *A* and *M* are close to the their boundaries, at least one of the terms *A*, 1−A, *M*, 1−M will be close to 0. Thus, at least one of the terms of $log^{2}\left(A\right)$, $log^{2}\left(1-A\right)$, $log^{2}\left(M\right)$, $log^{2}\left(1-M\right)$ will be large enough to prevent the parameter estimations to reach the boundaries.

#### 2.1.2. Hypothesis Testing

For each SNP, consider testing the hypothesis $R=R_{0}$. We are interesting in testing the null hypothesis $H_{0}:R_{0}=1$ versus alternative hypothesis $H_{1}:R_{0}\neq 1$. The Wald test measures the squared difference ${\hat{R}}_{MLE}-R_{0}$ weighted by the curvature of the log-likelihood function. The Wald statistic is calculated as follows:

$$\begin{array}{c} W=\frac{{\left({\hat{R}}_{MLE}-R_{0}\right)}^{2}}{var({\hat{R}}_{MLE})} \end{array},$$

where ${\hat{R}}_{MLE}$ is the penalized MLE of *R*, and $var({\hat{R}}_{MLE})$ is its variance estimated by the inverse of the expected information matrix evaluated at the maximum likelihood estimate. Under the null hypothesis, the Wald test statistic *W* follows an asymptotic $\chi^{2}$ distribution with one degree of freedom.

Score test assesses the statistical significance of the parameter based on the gradient of the likelihood function. The value of the score function is evaluated at $R_{0}$ and equaled to 0 when $R_{0}={\hat{R}}_{MLE}$. When the score function at $R_{0}$ deviates far from 0, the alternative hypothesis is more plausible than $H_{0}$. The Score test statistic is calculated as follows:

$$\begin{array}{c} S=\frac{{\left(u\left(R_{0}\right)\right)}^{2}}{I(R_{0})} \end{array},$$

where $u(R_{0})$ is the score function evaluated at $R_{0}$ and other parameters are replaced by the MLE, and $I(R_{0})$ is the Fisher information evaluated by $R_{0}$ while other parameters are fixed at their MLE. The test statistic asymptotically follows a $\chi^{2}$ distribution with one degree of freedom.

The likelihood ratio test is another method to assess statistical significance based on the comparison of log-likelihood function evaluated at MLE and $R_{0}$. The likelihood ratio test statistic is calculated as follows:

$$\begin{array}{c} LR=2l\left({\hat{R}}_{MLE}\right)-2l\left(R_{0}\right) \end{array},$$

where $l({\hat{R}}_{MLE})$ and $l(R_{0})$ is the log-likelihood function evaluated at ${\hat{R}}_{MLE}$ and $R_{0}$, respectively. Under large samples the likelihood ratio test statistic asymptotically follows a $\chi^{2}$ distribution with one degree of freedom.

### 2.2. Bayesian Hierarchical Modeling

The genome-wide association studies have effectively detected many SNPs that are associated with various diseases. However, the identified variants explain a limited portion of the disease heritability [5]. Many associated variants remain undiscovered partly because the single-locus GWAS has limited power to reveal the associations. For example, the *p*-values from the hypothesis tests outlined in Section 2.1 require adjustment for multiple testing using methods such as Bonferroni correction or False Discovery Rate (FDR) to control for false positives [9]. Only a limited number of markers with very large effect sizes can pass the stringent criteria after adjustments. In addition, the SNP interactions, which play an important role in complex diseases susceptibility [10], are ignored in single-marker analysis.

We propose a Bayesian hierarchical model to jointly examine a SNP set in order to improve the detection power. The literature contains numerous models for SNP-set tests, including the burden test [11], SKAT [12], aSPU test [13], VEGAS/VEGAS2 [14,15], MAGMA [16], GATES [17], GHC [18], GBJ [19], and ACAT tests [20]. However, existing SNP-set tests are designed for traditional GWAS where cases and controls are sampled independently. We consider paired tumor–normal specimens where tumor and normal tissues were collected from the same patient. Therefore, traditional GWAS-based tests are not directly applicable to the matched pair data.

The multiple marker model can aggregate a group of SNPs in a biologically meaningful way, such as genes, pathways, or topological 3D structures [21]. For simplicity, we use genes as an example of grouping SNPs, with a note that the model applies to any other biologically meaningful way of grouping SNPs. The multiple marker model is applied to determine the association status of a gene, after integrating data from all genetic markers located in the gene region.

#### 2.2.1. Prior Distribution

Suppose there are *J* SNPs on a gene. Let *G* denote the gene association status: G=1 if associated and 0 otherwise. Gene status *G* is assigned a Bernoulli prior with probability *b*, where *b* is further assigned a Beta hyper prior. Assuming only a small proportion of genes are associated, set the prior mean of *b* at a small value, e.g., 0.2. The distributions of *G* and probability *b* are as follows:

$$\begin{array}{cc} & f\left(G|b\right)=b^{G}\cdot {\left(1-b\right)}^{\left(1-G\right)},\\ & f\left(b\right)∼Beta\left(\alpha_{b},\beta_{b}\right). \end{array}$$

Let $H_{j}$, j=1,...,J, represent the association status of the *j*thSNP on the gene, where $H_{j}=1$ if the SNP is a risk marker, and $H_{j}=0$ if it is unrelated or neutral. We assume the probability of $H_{j}=1$ depend on the gene status *G*. If G=1, there is a high probability that the SNPs on the gene are associated with the disease. Otherwise, the probability is low. Let $H_{j}|\left(G=i\right)∼Bernoulli\left(p_{i}\right)$, i=0, 1. For example, we can set $p_{0}=0.1$ and $p_{1}=0.9$, but they can take other values to reflect the prior knowledge. Thus, the prior distribution of $H_{j}$ given *G* is

$$f\left(H_{j}|G\right)=p_{1}^{G}\cdot p_{0}^{\left(1-G\right)}.$$

Let $H=\{H_{1},\ldots ,H_{J}\}$. Assuming that each $H_{j}$ is conditionally independent, we can write the prior distribution as follows:

$$\begin{array}{c} f\left(H|G\right)=f\left(H_{1},\ldots ,H_{J}|G\right)=\prod_{j=1}^{J}f\left(H_{j}|G\right) \end{array}.$$

Let $R_{j}$ denote the relative risk (RR) of the *j*th SNP on the gene. The prior distribution of $R_{j}$ depends on the association status of the *j*th SNP. When it is a risk mutant, the effect size should be greater than one. We assign $R_{j}$ a $Gamma(k_{1},\theta_{1})$ distribution with the mean greater than one. In practice, the prior mean can be the average of all MLEs of the SNPs. On the other hand, when the *j*th SNP is neutral, the effect size should be one, and thus $R_{j}$ is assigned a prior $Gamma(k_{0},\theta_{0})$ with mean at one. Let $R=\{R_{1},\ldots ,R_{J}\}$ be a set of risk parameters on the same gene. Assume $R_{j}$ is conditionally independent of each other given H, and $R_{j}$ is independent of $H_{j^{\prime }}$ when $j\neq j^{\prime }$. Then the prior distribution of R is as follows:

$$\begin{array}{cc} f(R_{j}|H_{j}) & =\gamma {\left(k_{1},\theta_{1}\right)}^{H_{j}}\cdot \gamma {\left(k_{0},\theta_{0}\right)}^{\left(1-H_{j}\right)},\\ f(R|H) & =\prod_{j=1}^{J}f\left(R_{j}|H_{j}\right), \end{array}$$

where $\gamma (k_{1},\theta_{1})$ and $\gamma (k_{0},\theta_{0})$ are the density function of $Gamma(k_{1},\theta_{1})$ and $Gamma(k_{0},\theta_{0})$, respectively.

Let $A_{j}$ and $M_{j}$ denote the risk allele frequency (AF) and the mutation rate (MR), respectively, of the *j*th SNP on the gene. The prior distribution of $A_{j}$ depends on the association status of the *j*th SNP. The observed and expected AF are

(2) $$\begin{array}{cc} {\hat{A}}_{j} & =\frac{n_{10}+n_{11}+n_{12}+2n_{20}+2n_{21}+2n_{22}}{2n},\\ \mu_{{\hat{A}}_{j}}=E\left({\hat{A}}_{j}\right) & =E\left(\frac{n_{10}+n_{11}+n_{12}+2n_{20}+2n_{21}+2n_{22}}{2n}\right)\\ \; & =\frac{A_{j}\left(R_{j}-A_{j}+R_{j}A_{j}+2A_{j}M_{j}-2R_{j}A_{j}M_{j}+1\right)}{2(R_{j}A_{j}-M_{j}-A_{j}+R_{j}M_{j}+2A_{j}M_{j}-2R_{j}A_{j}M_{j}+1)}. \end{array}$$

When the SNP is neutral, i.e., $R_{j}=1$, $E\left({\hat{A}}_{j}\right)=A_{j}$. Thus $A_{j}|\left(H_{j}=0\right)$ is assigned a $Beta(\alpha_{A0,j},\beta_{A0,j})$ prior distribution with the mean set at ${\hat{A}}_{j}$. On the other hand, for a risky SNP, the allele frequency is enriched in the patient population. We can estimate the parameter $A_{j}$ by solving the equation ${\hat{A}}_{j}=\mu_{{\hat{A}}_{j}}$:

(3) $$\begin{array}{c} A_{j}={\hat{A}}_{j}+\frac{R_{j}+1-\sqrt{{\left[R_{j}+{\hat{A}}_{j}2\left(2M_{j}-1\right)\left(R_{j}-1\right)+1\right]}^{2}-8{\hat{A}}_{j}\left(2M_{j}-1\right)\left(R_{j}-1\right)\left(R_{j}M_{j}-M_{j}+1\right)}}{2\left(2M_{j}-1\right)\left(R_{j}-1\right)}. \end{array}$$

In Equation (3) the parameters $R_{j}$ and $M_{j}$ are unknown, and they can be set to the MLE or other estimates. Then, $A_{j}|H_{j}=1$ is assigned a $Beta(\alpha_{A1,j},\beta_{A1,j})$ distribution, with the prior mean equal to (3). Let $A=\{A_{1},\ldots ,A_{J}\}$ be a set of AF variables on the same gene. Assume that $A_{j}$ is conditionally independent of each other given H, and $A_{j}$ is independent of $H_{j^{\prime }}$ when $j\neq j^{\prime }$. Therefore, the prior distribution of A is as follows:

$$\begin{array}{cc} f(A_{j}|H_{j}) & =\beta {\left(\alpha_{A1,j},\beta_{A1,j}\right)}^{H_{j}}\cdot \beta {\left(\alpha_{A0,j},\beta_{A0,j}\right)}^{\left(1-H_{j}\right)},\\ f(A|H) & =\prod_{j=1}^{J}f\left(A_{j}|H_{j}\right), \end{array}$$

where $\beta (\alpha_{A1,j},\beta_{A1,j})$ and $\beta (\alpha_{A0,j},\beta_{A0,j})$ are the density function of $Beta(\alpha_{A1,j},\beta_{A1,j})$ and $Beta(\alpha_{A0,j},\beta_{A0,j})$, respectively.

The prior distribution of MR parameter $M_{j}$ depends on the association status of the *j*th SNP. The observed and expected mutation rate from the sample are

(4) $$\begin{array}{cc} {\hat{M}}_{j} & =\frac{n_{01}+2n_{02}+n_{10}+n_{12}+2n_{20}+n_{21}}{2n},\\ \mu_{{\hat{M}}_{j}}=E\left({\hat{M}}_{j}\right) & =E\left(\frac{n_{01}+2n_{02}+n_{10}+n_{12}+2n_{20}+n_{21}}{2n}\right)\\ \; & =\frac{M_{j}\left(R_{j}-M_{j}+R_{j}M_{j}+2A_{j}M_{j}-2R_{j}A_{j}M_{j}+1\right)}{2(R_{j}A_{j}-M_{j}-A_{j}+R_{j}M_{j}+2A_{j}M_{j}-2R_{j}A_{j}M_{j}+1)}. \end{array}$$

When the SNP is non-associated, $E\left(M_{j}|H_{j}=0\right)=M_{j}$. The MR is thus assigned a prior $Beta(\alpha_{M0,j},\beta_{M0,j})$. For a risky SNP, an estimate of $M_{j}$ can be obtained by letting ${\hat{M}}_{j}=\mu_{{\hat{M}}_{j}}$ and then solving the equation:

(5) $$\begin{array}{c} M_{j}={\hat{M}}_{j}+\frac{-\left(R_{j}+1\right)+\sqrt{{\left[R_{j}+1-2{\hat{M}}_{j}\left(R_{j}-1\right)\left(1-2A_{j}\right)\right]}^{2}+8{\hat{M}}_{j}\left(1-2A_{j}\right)\left(R_{j}-1\right)\left(R_{j}A_{j}-A_{j}+1\right)}}{2\left(R_{j}-1\right)\left(1-2A_{j}\right)}. \end{array}$$

We can assign a $Beta(\alpha_{M1,j},\beta_{M1,j})$ prior for $M_{j}$ conditional on $H_{j}=1$, where the prior mean is equal to (5). Let $M=\{M_{1},\ldots ,M_{J}\}$. Assume that $M_{j}|H_{j}$ is independent of each other, and $M_{j}$ is independent of $H_{j^{\prime }}$ when $j\neq j^{\prime }$. Therefore, the distribution of M is as follows:

$$\begin{array}{cc} f(M_{j}|H_{j}) & =\beta (\alpha_{M1,j},\beta_{M1,j})\cdot \beta {\left(\alpha_{M0,j},\beta_{M0,j}\right)}^{\left(1-H_{j}\right)},\\ f(M|H) & =\prod_{j=1}^{J}f\left(M_{j}|H_{j}\right). \end{array}$$

#### 2.2.2. Joint Posterior Distribution

Let $\Theta_{j}=\left\{R_{j},A_{j},M_{j}\right\}$ be the set of RR, AF and MR parameters on the *j*th SNP, where $\Theta_{j}$ and $\Theta_{j^{\prime }}$ are independent when $j\neq j^{\prime }$. Let $\Theta =\{R_{1},...,R_{J},A_{1},...,A_{J},M_{1},...,M_{J}\}$ be the set of RR, AF, and MR parameters on all SNPs of a given gene. Let $H=\{H_{1},...,H_{J}\}$ be a set of all SNP association status on then gene. Under the assumption that $R_{j}|H_{j},\;A_{j}\left|H_{j},\;M_{j}\right|H_{j}$ are independent of each other, the joint distribution of Θ,H,G,b can be derived as follows:

$$\begin{array}{cc} f(\Theta ,H,G,b) & =\left(\prod_{j=1}^{J}f\left(R_{j}|H_{j}\right)f\left(A_{j}|H_{j}\right)f\left(M_{j}|H_{j}\right)\;f\left(H_{j}|G\right)\right)\;f\left(G|b\right)\;f\left(b\right). \end{array}$$

The observed counts of the normal–tumor paired genotypes at the *j*th SNP, defined as $n_{j}=\left\{n_{00(j)},n_{01(j)},n_{02(j)},n_{10(j)},n_{11(j)},n_{12(j)},n_{20(j)},n_{21(j)},n_{22(j)}\right\}$, follows a Multinoulli distribution with 9 categories, where the expected probabilities are shown in (1). Let $S=\{n_{1},...,n_{J}\}$ be the set of counts on a given gene. Assume conditional independence of $n_{j}$ and $n_{j^{\prime }}$ when $j\neq j^{\prime }$. The likelihood function is as follows:

$$\begin{array}{cc} f(S|\Theta ) & =\prod_{j=1}^{J}\prod_{k,k^{\prime }}^{0,1,2}P_{k,k^{\prime }\left(j\right)}^{n_{k,k^{\prime }\left(j\right)}} \end{array}\;.$$

Therefore, the joint posterior distribution can be derived as follows:

$$f(\Theta ,H,G,b|S)\propto f\left(S|\Theta \right)\;\left(\prod_{j=1}^{J}f\left(R_{j}|H_{j}\right)f\left(A_{j}|H_{j}\right)f\left(M_{j}|H_{j}\right)\;f\left(H_{j}|G\right)\right)\;f\left(G|b\right)\;f\left(b\right).$$

The posterior distributions lack closed-form expressions, and thus it is difficult to sample from the posterior distributions directly. We estimate them using Markov Chain Monte Carlo (MCMC) simulation. For parameters having a closed-form conditional posterior distribution, such as (G,b,H), a Gibbs sampler is used to approximate the target distribution. Other parameters are sampled with the Metropolis–Hastings algorithm in each Gibbs iteration. Details can be found in the Supplementary Materials.

## 3. Results

### 3.1. Simulation Studies

We evaluate penalized MLE, single-marker Bayesian (J=1), and multi-marker Bayesian models using simulated paired data with varying (1) sample sizes (n=1000, 3000); (2) allele frequencies (*A* = 0.05, 0.1, 0.2); (3) mutation rates (*M* = 0.001, 0.005); and (4) relative risks (*R* = 1, 2, 3). Each setting is repeated 100 times. We focus on the estimation accuracy of the relative risk *R*, which is the major parameter of interest. For hypothesis testing, we compare the type I error rates under the null hypothesis $H_{0}$: *R* = 1, and the power to identify associated variants under the alternative hypotheses $H_{a}$: *R* = 2 and $H_{a}$: *R* = 3. The allelic risk model is assumed to be additive, where the risk of the heterozygous genotype is the additive mean of the two homozygous genotypes [22].

For multi-marker analysis, we consider a gene that has 4 SNPs having the same relative risk. To compare the performance of estimating the *R*, we use the mean square error (MSE) for penalized MLE, the single-marker Bayesian model and the multi-marker Bayesian model. For Bayesian models we use the sample median of the posterior draws. In the penalized MLE method, we let the ridge coefficient = 0.05 to regularize the estimation.

In Bayesian modeling, the prior distributions for an unassociated SNP are set as follows: R|(H=0)∼Gamma(3,3); A|(H=0) follows a Beta distribution with mean at the observed allele frequency; M|(H=0) follows a Beta distribution with mean at observed mutation rate. The prior distributions conditioning on H=1 are: R|(H=1)∼Gamma(3,8); A|(H=1) follows a Beta distribution with the mean chosen according to (3); M|(H=1) follows a Beta distribution whose mean equals to (5). MCMC simulations with three restarts are used to draw from the posterior distribution for model parameters. The Bayesian estimation is derived by the median of MCMC samples after burn-in and thinning.

**Figure 1:**
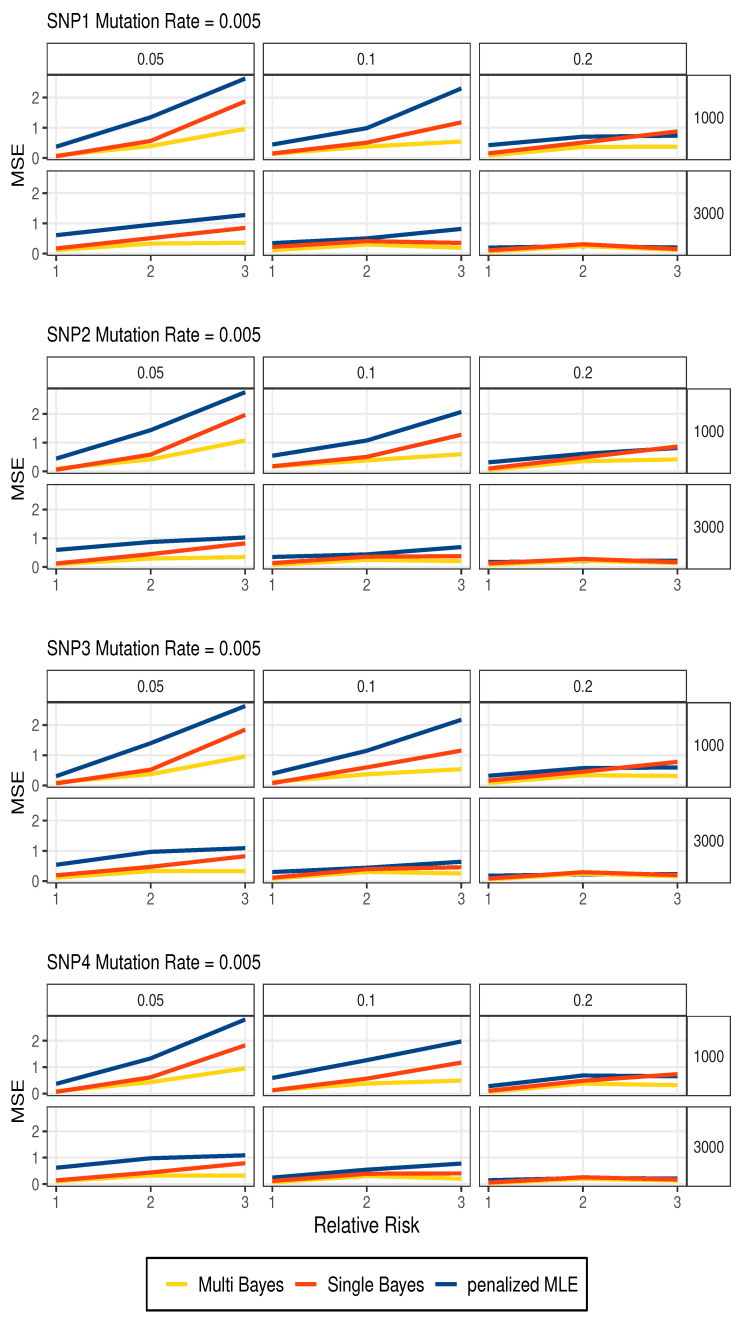
MSE of penalized MLE, single-marker Bayesian and multi-marker Bayesian model (*M* = 0.005).

Figure 1 shows the MSE of estimators over 100 replicates when M=0.005. The results show that multiple-marker Bayesian model has the lowest MSE in most settings, especially under small sample sizes or low mutation rates. Similar results are observed for M=0.001 (Supplementary Materials).

Next we consider the hypothesis testing. To estimate the false positive rate (type I error), we simulate data from the null model that no SNPs are associated with the disease phenotype, i.e., all SNP-level association are H=0. In this situation, all the SNP relative risks are set to R=1. To estimate the power (True Positive Rate), we simulate data under the alternative hypothesis that all SNP-level association are H=1. To vary the association level, we considered two scenarios, R=2 and 3. We fix the nominal type I error rate at α=0.05 for the likelihood-based tests. All three tests (Wald, Score, likelihood ratio) have similar performance, and we choose the Wald test as a representative under the penalized MLE method. In single- and multi-marker Bayesian models, we use the posterior median of *H* to make a decision for the association of the marker. Table 1 shows the estimated type I error and the power based on 100 replicates of the three methods when M=0.005. When the sample size is 1000 and the allele frequencies are low, all methods fail to control the type I error rate at 0.05. However, the type I error of multi-marker Bayesian model is the lowest among all three methods in most settings. In moderate-risk case (R=2), the power of the multi-marker Bayesian either exceeds or is similar to the single-marker Bayesian model in all settings. When *R* increases from 2 to 3, the multi-marker Bayesian model shows a substantial power improvement for *n* = 1000. All performances are similar in settings with large sample size (*n* = 3000) and high-risk allele frequency. Similar results are observed for M=0.001 (Supplementary Materials).

Overall, the multi-marker Bayesian model outperforms the other two methods in most settings. The Bayesian model provides a more stable estimation than penalized MLE. In scenarios where data are limited due to insufficient sample size, low allele frequencies or low mutation rates, the multi-marker Bayesian model has a clear advantage over others.

### 3.2. Real Data Application

#### 3.2.1. Application to Matched-Pair Breast Cancer Data

We analyzed the tumor–normal matched-pair data for breast cancer from The Cancer Genome Atlas (TCGA). The total number of SNPs is 905,461 and the sample size is 1070. We applied quality control methods to remove invalid SNP genotypes. First, Hardy–Weinberg equilibrium test [23] was applied and *p*-value = 0.05 was used as the cut-off threshold. SNPs with missing genotype and the allele frequency less than 0.05 are removed. After quality control, we retained 614,883 SNPs.

**Figure 2:**
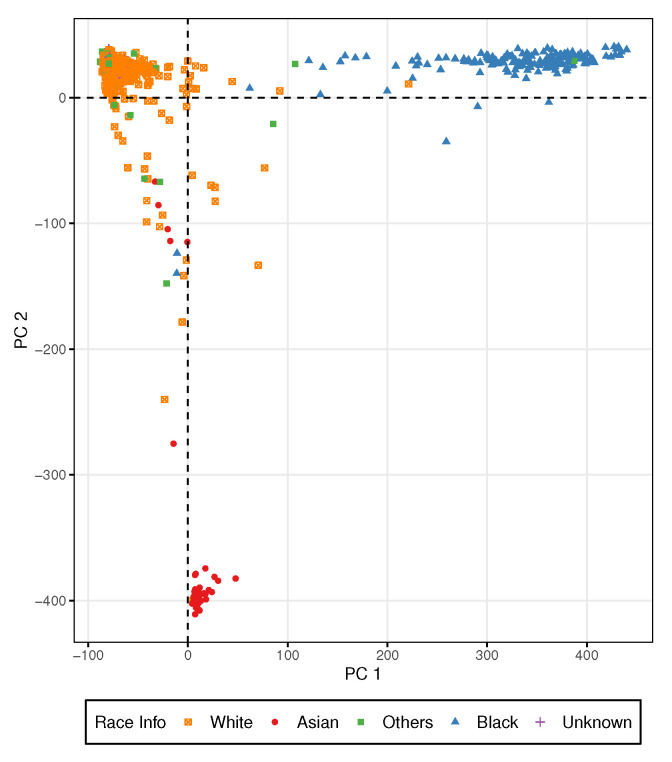
Principal Component Analysis of all input SNPs in the breast cancer data.

Among the 1070 samples, 725 are self-reported as White, 176 are Black, 60 are Asians, 95 are Others, and 109 are Unknown. We applied the principal Component Analysis (PCA) using all input SNPs to determine the patient population structure. Ethnicity groups were distinguishable using the top two principal components as shown in Figure 2. We imputed the missing racial ancestry for unknown patients. To avoid potential problems caused by population stratification, we used 807 samples classified as White in the subsequent analysis.

We used the refFlat gene annotation (UCSC hg19) for human genome references. The gene region is determined using transcription start and end positions. A SNP is on a gene region if it locates within 1000 base pairs upstream or downstream of a gene. A total of 58,545 genes are available, among which 20,353 contain at least one SNP, with a total of 220,268 SNPs being mapped to these genes. The summary statistics about SNP counts and gene length (in base pairs) of all genes are provided in Table 2.

The SNPs tend to be widely separated across a long gene region, where linkage disequilibrium can hardly be observed. To capture the joint effects of adjacent markers, we split long genes evenly into small segments that contain 32,000 base pairs or less. After segmentation, there were 58,161 gene segments. Summary statistics about SNP counts on each gene segment is given in Table 3. Then, multi-marker analysis method was applied to all 58,161 gene segments to analyze the joint effects.

#### 3.2.2. Single-Marker Analysis

We applied the Bayesian single-marker analysis to all 220,268 SNPs. The individual SNP status is estimated by the mean of the posterior distribution. To obtain aggregated scores for genes, we first computed the average of all SNPs on a gene segment, and then pick the largest segment to represent the gene.

Table 4 shows top ranked genes identified by the single-marker Bayesian model. Among them, many have been reported to be cancer related. Multiple recent studies have indicated a positive correlation between boosted TIAM1 expression level and higher grade of human breast cancer [24,25]. The TIAM1 gene and the encoded protein have been implicated in cell proliferation, migration, invasion, and tumor progression in a variety of human cancer [26,27,28,29]. Genetic loss of NDST4 is significantly associated with tumor progression, and NDST4 gene is identified as a novel candidate tumor suppressor in human colorectal cancer [30]. A number of studies have suggested that the deactivation of EIF2AK2 can suppress tumor growth [31,32,33], while elevated expression of EIF2AK2 increases carcinoma progression in a variety of human cancer, including breast cancer [34,35,36]. The TMEM117 gene belongs to the TMEM family. There is evidence that down- or up-regulated TMEM expression has been identified in tumor tissues compared to adjacent healthy tissues, and some suggest TMEMs as prognostic biomarkers [37].

#### 3.2.3. Multi-Marker Analysis

The multi-marker Bayesian model was also applied to the TCGA breast cancer data. In this results, the gene segment status is estimated by the median of the posterior distribution. Similarly, gene status is represented by the maximum value of all gene segments.

Table 5 shows the top genes identified in the multi-marker Bayesian analysis. Recent studies have reported a highly significant association between the KIRREL3 region and breast cancer [38]. The STX3 gene may contribute to carcinogenesis via up- or down-regulation in various cancer, promoting breast cancer cell growth [39,40]. Elevated AGPAT4 expression in cancer tissues is associated with poorer survival rates in colorectal cancer patients [41]. Deletions in the PKNOX2 gene region are linked to breast cancer and ovarian cancer malignancies [42,43]. The RGS3 protein may function as a tumor suppressor [44]. Low expression of CSMD1, a tumor suppressor gene, is significantly associated with higher breast tumor grades [45,46].

**Figure 3:**
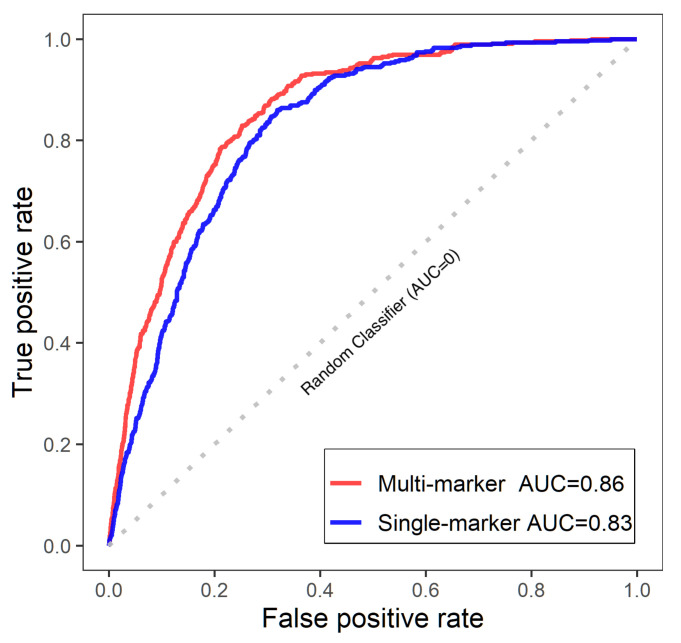
ROC curves of multiple and single marker models. Dotted line: Random Classifier (AUC = 0).

We used the external oncogenic database, the Catalogue Of Somatic Mutations In Cancer (COSMIC), which provides comprehensive somatic mutations and genes that are associated with all types of breast cancer tissues. The gene list contains gene symbols, mutated samples, and total samples. The COSMIC breast cancer genes are sorted by mutated rates. Genes with higher mutation rates tend to have greater risk in breast cancer. The top 500 genes from COSMIC were used as benchmark to compare with top ranked genes of multi- and single-marker analysis. We plotted the receiver operating characteristic (ROC) curves in Figure 3, and calculated the area under curve (AUC) of both methods. The AUC of multi-marker analysis is 0.86 and the AUC of single-marker analysis is 0.83.

**Figure 4:**
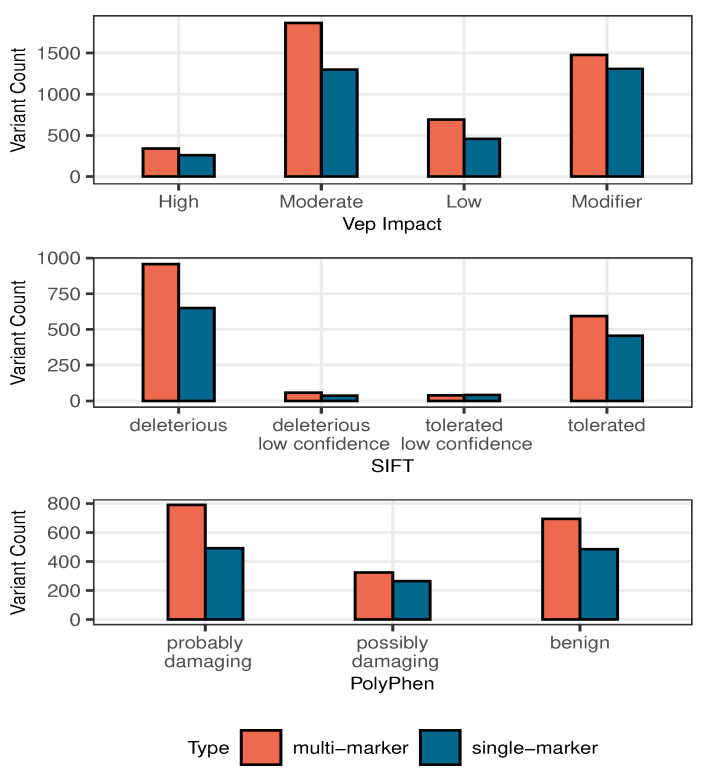
The counts of impact levels predicted by three tools: Ensembl VEP, SIFT and PolyPhen using the variants from the top 100 associated genes identified by multiple-marker Bayesian model and single-marker Bayesian model.

We also explored external resources from the Genomic Data Commons (GDC) Data Portal to compare with the gene lists generated by our Bayesian models. The GDC data include mutations reported to be associated with breast cancer. The impact of these mutations is classified based on the severity of the variant consequences using three tools: Ensembl Variant Effect Predictor (VEP), Polymorphism Phenotyping (PolyPhen) and Sorting Intolerant From Tolerant (SIFT). The Ensembl VEP tool evaluates the effect of a genomic variant in coding and non-coding regions. The effect levels include high, moderate, low, and modifier, which range from high impact in protein to no evidence of impact. The SIFT tool predicts whether an amino acid substitution will affect protein function and phenotype based on sequence homology. The impact levels are “deleterious”, “deleterious low confidence”, “tolerated low confidence” and “tolerated”, which ranges from very likely to not likely to have a phenotypic effect. The PolyPhen tool predicts the potential impact of an amino acid substitution on human proteins. The impact levels are “probably damaging”, “possibly damaging”, “benign”, and “unknown”, ranging from high confidence of affecting protein function or structure to an indeterminate prediction. In Figure 4, we summarized the counts of each impact level for variants within the top 100 associated genes identified by single-marker and multi-marker Bayesian models. The results indicate that variants identified by the multi-marker model are more frequently classified as high impact compared to those identified by the single-marker model.

## 4. Discussion

Studies have indicated that the progression of cancer is associated with the accumulation of somatic mutations [8,47]. Investigating the impact of somatic mutations in carcinoma is critical in risk prediction, continuous monitoring, and early detection of cancer, and can contribute to individualized prevention and therapeutic strategies. We proposed a novel model framework to analyze somatic mutations using tumor and matched normal tissue data in GWAS. The penalized maximum likelihood estimation (MLE) provides a computationally efficient method for individual SNP analysis. The single-marker hierarchical Bayesian model, compared to the penalized MLE method, has low MSE in the relative risk estimation and high power to identify associated SNPs with limited sample size, low allele frequency or low mutation rates. However, in settings with sufficient sample size, high allele frequency and mutation rate, the performance of penalized MLE and single-marker Bayesian method are similar. The multi-marker hierarchical Bayesian model groups SNPs into biologically meaningful sets, allowing joint analysis of their effects. In the breast cancer data analysis, large genes were divided into smaller segments (~32,000 base pairs or less), with the multi-marker Bayesian model applied to each segment comprising localized relevance sets of 2–40 SNPs (Section 3.2.1). Simulations consistently demonstrated that this model maintains a low type I error rate and enhances power (TPR) compared to single-marker or penalized MLE methods, particularly for moderate effect sizes. Additionally, the algorithm substantially reduces the multiple testing burden, decreasing the number of tests from 220,268 SNPs to 20,353 genes. It is worth mentioning that the cost of computation is similar for multiple- and single-marker Bayesian model. Future work will focus on extending the model to incorporate interaction networks and applying it to other cancer types.

## Figures and Tables

**Table 1:** Estimated type I error rates (*R* = 1) and power (*R* = 2, 3) of penalized MLE, single-marker Bayesian and multi-marker Bayesian model (*M* = 0.005).

*n*	SNP	Allele	Multi Bayes			Single Bayes			Penalized MLE		
		**Freq**	**R = 1**	**R = 2**	**R = 3**	**R = 1**	**R = 2**	**R = 3**	**R = 1**	**R = 2**	**R = 3**
1000	SNP1	0.05	0.14	0.31	0.64	0.24	0.32	0.45	0.26	0.13	0.09
		0.10	0.14	0.38	0.82	0.23	0.36	0.59	0.18	0.10	0.21
		0.20	0.07	0.39	0.91	0.18	0.34	0.65	0.18	0.09	0.55
	SNP2	0.05	0.14	0.31	0.62	0.23	0.31	0.44	0.30	0.16	0.12
		0.10	0.15	0.39	0.81	0.25	0.38	0.57	0.22	0.13	0.18
		0.20	0.07	0.40	0.91	0.15	0.35	0.67	0.18	0.10	0.55
	SNP3	0.05	0.14	0.32	0.64	0.23	0.34	0.47	0.26	0.12	0.14
		0.10	0.14	0.36	0.82	0.21	0.32	0.59	0.20	0.11	0.20
		0.20	0.07	0.41	0.92	0.18	0.37	0.67	0.15	0.09	0.53
	SNP4	0.05	0.14	0.31	0.64	0.24	0.31	0.47	0.19	0.12	0.11
		0.10	0.14	0.38	0.83	0.23	0.35	0.59	0.21	0.13	0.15
		0.20	0.07	0.44	0.92	0.15	0.42	0.70	0.19	0.15	0.58
3000	SNP1	0.05	0.12	0.45	0.91	0.22	0.38	0.68	0.24	0.04	0.17
		0.10	0.08	0.55	0.98	0.19	0.44	0.84	0.15	0.17	0.80
		0.20	0.05	0.62	0.99	0.12	0.47	0.91	0.11	0.49	0.99
	SNP2	0.05	0.11	0.44	0.91	0.20	0.38	0.67	0.18	0.03	0.14
		0.10	0.06	0.55	0.98	0.14	0.42	0.84	0.16	0.13	0.82
		0.20	0.05	0.62	0.99	0.13	0.46	0.90	0.18	0.42	1.00
	SNP3	0.05	0.12	0.44	0.91	0.23	0.40	0.68	0.12	0.05	0.16
		0.10	0.06	0.55	0.97	0.12	0.44	0.82	0.16	0.12	0.83
		0.20	0.04	0.64	0.99	0.10	0.51	0.90	0.09	0.50	0.99
	SNP4	0.05	0.11	0.46	0.91	0.19	0.42	0.69	0.18	0.02	0.12
		0.10	0.06	0.55	0.98	0.14	0.44	0.82	0.15	0.15	0.77
		0.20	0.03	0.62	0.99	0.09	0.45	0.90	0.18	0.48	1.00

**Table 2:** Summary of SNP counts, gene length on all genes.

Summary	Min	Q1	Median	Mean	Q3	Max
**SNP Counts**	1	3	8	42	39	1242
**Gene Length**	21	16,215	47,282	159,292	166,206	$2.32\times 10^{6}$

**Table 3:** Summary of SNP counts on gene segments.

Summary	Min	Q1	Median	Mean	Q3	Max
**SNP counts**	2	3	5	6	8	40

**Table 4:** Genes with highest gene status estimated by posterior median in single-marker analysis.

Gene Name	Chr	Gene Status
IL7	chr8	0.975
TIAM1	chr21	0.972
CKAP2L	chr2	0.961
TTC28	chr22	0.956
NDST4	chr4	0.952
EIF2AK2	chr2	0.952
CACNB4	chr2	0.95
PARD3B	chr2	0.946
TMEM117	chr12	0.944
ATP6V0D1	chr16	0.943

**Table 5:** Genes with highest gene status estimated by posterior median in multiple-marker analysis.

Gene Name	Chr	Gene Status
LINC00383	chr13	0.999
KIRREL3	chr11	0.999
STX3	chr11	0.999
AGPAT4	chr6	0.999
SYCE1	chr10	0.997
RCBTB1	chr13	0.997
PKNOX2	chr11	0.997
RGS3	chr9	0.997
GCSH	chr16	0.997
CSMD1	chr8	0.996

## Data Availability

R code available at https://github.com/bysKate/matchedGWAS (accessed on 10 October 2025).

## Supplementary Materials

The following supporting information can be downloaded at https://www.mdpi.com/article/10.3390/e27101077/s1, Figure S1: MSE of penalized MLE, Single-marker and Multiple-marker Bayesian models (*M* = 0.001); Figure S2: Estimated type I error rates (*R* = 1) and power (*R* = 2, 3) of penalized MLE, Single-marker and Multiple-marker Bayesian models (*M* = 0.001); Figure S3: Estimated relative risk, segment status, and SNP counts of 10 segments on TACC2 gene; Figure S4: Estimated relative risk, segment status, and SNP counts of 71 segments on CSMD1 gene; Figure S5: Estimated relative risk, segment status, and SNP counts of 41 segments on CDH13 gene. References [48,49,50] are citied in the Supplementary Materials.
