# Supplementary material for: A Bayesian Model for Paired Data in Genome-Wide Association Studies with Application to Breast Cancer
Source: Entropy (Basel). 2025 Oct 18;27(10):1077. doi: 10.3390/e27101077 (PMC12562651; doi:10.3390/e27101077)
Supplement: Supplementary file 1 [file entropy-27-01077-s001.zip › entropy-3822789-supplementary.pdf]

Supplementary Materials

A Bayesian Model for Paired Data in Genome-Wide Association Studies with Application to Breast Cancer

1 Muti-Marker Bayesian Model

1.1 Posterior sampling

The posterior distributions do not have closed-form expressions, making direct sampling challenging. We employ Markov Chain Monte Carlo (MCMC) methods to estimate these distributions. For parameters with closed-form conditional posterior distributions, such as $(G, b, \mathbf{H})$, we use a Gibbs sampler to approximate the target distribution. Other parameters are sampled using the Metropolis-Hastings algorithm within each Gibbs iteration. For a gene with J SNPs, the Markov Chain Monte Carlo simulation proceeds as follows:

1. Initialization of parameters.
   Assign initial values to $b^0, G^0, \mathbf{H^0}, \mathbf{R^0}, \mathbf{A^0}, \mathbf{M^0}$ at the beginning of simulation. The MCMC simulates 3 chains and assigns different initial values for different chains and parameters.

2. Update b^t.
   Given other parameters are fixed, the conditional posterior distribution is derived as follows:
   $$f(b^t|\mathbf{S}, G^t, \mathbf{H^t}, \mathbf{\Theta^t}) \propto (b^t)^{G^t+\alpha_b-1}(1-b^t)^{\beta_b-G^t}.$$
   The right hand side is a Beta distribution. Update b^t by sampling from the Beta distribution.

3. Update G^t.
   $$f(G^t|\mathbf{S}, b^t, \mathbf{H^t}, \mathbf{\Theta^t}) \propto f(\mathbf{H^t}|G^t)f(G^t|b^t)$$

Substitute with the previous equation, there are

$$f(G^t|\mathbf{S}, b^t, \mathbf{H^t}, \mathbf{\Theta^t}) \propto \left[b^t p_1^{\sum H_j^t}(1-p_1)^{J-\sum H_j^t}\right]^{G^t} \cdot \left[(1-b^t)\cdot p_0^{\sum H_j^t}(1-p_0)^{J-\sum H_j^t}\right]^{(1-G^t)}$$

The conditional posterior probability density function of G^t is proportional to a Bernoulli distribution. Then G^{t+1} can be sampled from Gibbs sampler based on current state:

$$G^{t+1} \sim Bernoulli\left(\frac{b^t p_1^{\sum H_j^t}(1-p_1)^{J-\sum H_j^t}}{b^t p_1^{\sum H_j^t}(1-p_1)^{J-\sum H_j^t} + (1-b^t)\cdot p_0^{\sum H_j^t}(1-p_0)^{J-\sum H_j^t}}\right)$$

4. Update $\mathbf{H} = (H_1, H_2, ..., H_J)$.
   When $H_j = 1$, the jth SNP is associated. Otherwise the jth SNP is not associated. Given other parameters are fixed, the conditional posterior distribution at step t can be derived as follows:

$$\begin{aligned} &f(H_j^t|\mathbf{S}, b^t, G^t, H_1^t, ..., H_{j-1}^t, H_{j+1}^t, ..., H_J^t, \mathbf{\Theta^t}) \\ &\propto f(R_j^t|H_j^t)f(A_j^t|H_j^t)f(M_j^t|H_j^t)f(H_j^t|G^t) \end{aligned}$$

   It can be shown that the conditional posterior distribution of H_j^t is proportional to a Bernoulli distribution.

5. Update relative risk $\mathbf{R^t} = R_1^t, R_2^t, ..., R_J^t$.
   Parameters are updated one by one in the order $R_1^0, R_2^0, ..., R_J^0, R_1^1, R_2^1, ..., R_J^1, ..., R_1^t, R_2^t, ..., R_J^t, R_1^{t+1}, R_2^{t+1}, ..., R_J^{t+1}$, conditioning on all other parameters being fixed. Since there is no closed form for conditional posterior distribution, the desired conditional posterior distribution will be sampled by Metropolis-Hastings algorithm within each Gibbs step.

   (a) Conditional posterior distribution.
   The proportional posterior distribution of R_j^t conditional on other parameters is derived as follows:

$$\begin{aligned} &f(R_j^t|\mathbf{S}, b^t, G^t, \mathbf{H^t}, R_1^t, ..., R_{j-1}^t, R_{j+1}^t, ..., R_J^t, \mathbf{A^t}, \mathbf{M^t}) \\ &\propto f(\mathbf{n_j^t}|R_j^t, A_j^t, M_j^t)f(R_j^t|H_j^t) \end{aligned}$$

   The marginal distribution can hardly be integrated. Thus the Metropolis-Hastings algorithm is applied.

   (b) Proposal distribution.
   A Gamma distribution is used as the proposal distribution to generate a candidate

value R_j^* in the neighborhood based on the value of current state R_j^t. The transition probability and reverse transition probability are as follows:

$$q(R_j^*|R_j^t) = Gamma(shape = 1 + 5R_j^t, rate = 5)$$

$$q(R_j^t|R_j^*) = Gamma(shape = 1 + 5R_j^*, rate = 5)$$

(c) Acceptance probability.

Compute the acceptance ratio and determine the acceptance probability:

$$acceptance\ ratio = \frac{f(\mathbf{n_j^t}|R_j^*, A_j^t, M_j^t)f(R_j^*|H_j^t) \cdot q(R_j^t|R_j^*)}{f(\mathbf{n_j^t}|R_j^t, A_j^t, M_j^t)f(R_j^t|H_j^t) \cdot q(R_j^*|R_j^t)}$$

Then compute the acceptance probability:

$$r = min\left\{1, \frac{f(\mathbf{n_j^t}|R_j^*, A_j^t, M_j^t)f(R_j^*|H_j^t) \cdot q(R_j^t|R_j^*)}{f(\mathbf{n_j^t}|R_j^t, A_j^t, M_j^t)f(R_j^t|H_j^t) \cdot q(R_j^*|R_j^t)}\right\}$$

The acceptance indicator is generated from $Bernoulli(r)$ distribution with probability equals r. If acceptance indicator equals to 1, the next state $R_j^{*1} = R_j^*$, otherwise $R_j^{*1} = R_j^t$.

(d) Iterative updates.

For Metropolis-Hastings iterations $h = 1, 2, ..., 99$, take R_j^{*h} as the current state, repeat the above steps to update the next state in Metropolis-Hastings sequences $R_j^{*1}, R_j^{*2}, ..., R_j^{*100}$. Finally, the next state of Gibbs iterations R_j^{t+1} is updated with the last state in Metropolis-Hastings iterations:

$$R_j^{t+1} = R_j^{*H}\ where\ H\ is\ the\ last\ state\ in\ MH\ iterations$$

6. Update allele frequency $\mathbf{A^t} = A_1^t, A_2^t, ..., A_J^t$.

Parameters are updated one by one in the order $A_1^0, A_2^0, ..., A_J^0$, $A_1^1, A_2^1, ..., A_J^1 ..., A_1^t, A_2^t, ...,$ $A_J^t, A_1^{t+1}, A_2^{t+1}, ..., A_J^{t+1}$, conditional on all other parameters are fixed. Within each Gibbs iteration, the Metropolis-Hastings algorithm proposes and updates the parameter for 100 times. Then the next Gibbs iteration is chosen from last 10 states of Metropolis-Hastings iterations.

(a) Conditional posterior distribution.

The proportional posterior distribution of A_j^t conditional on other parameters is derived as follows:

$$f(A_j^t|\mathbf{S}, b^t, G^t, \mathbf{H^t}, \mathbf{R^t}, A_1^t, ..., A_{j-1}^t, A_{j+1}^t, ..., A_J^t, \mathbf{M^t})$$
$$\propto f(\mathbf{n_j^t}|R_j^t, A_j^t, M_j^t) f(A_j^t|H_j^t)$$

The Metropolis-Hastings algorithm is applied.

(b) Proposal distribution.
Beta distribution is used as the proposal distribution to generate a candidate value A_j^* in the neighborhood based on the value of current state A_j^t. The transition probability and reverse transition probability are as follows:

$$q(A_j^*|A_j^t) = Beta(\alpha = \frac{1 + 10A_j^t}{1 - A_j^t}, \beta = 10)$$
$$q(A_j^t|A_j^*) = Beta(\alpha = \frac{1 + 10A_j^*}{1 - A_j^*}, \beta = 10)$$

(c) Acceptance probability.
Compute the acceptance ratio and determine the acceptance probability:

$$acceptance\ ratio = \frac{f(\mathbf{n_j^t}|R_j^t, A_j^*, M_j^t) f(A_j^*|H_j^t) \cdot q(A_j^t|A_j^*)}{f(\mathbf{n_j^t}|R_j^t, A_j^t, M_j^t) f(A_j^t|H_j^t) \cdot q(A_j^*|A_j^t)}$$

Then acceptance probability is determined as:

$$r = min\left\{1, \quad \frac{f(\mathbf{n_j^t}|R_j^t, A_j^*, M_j^t) f(A_j^*|H_j^t) \cdot q(A_j^t|A_j^*)}{f(\mathbf{n_j^t}|R_j^t, A_j^t, M_j^t) f(A_j^t|H_j^t) \cdot q(A_j^*|A_j^t)}\right\}$$

The acceptance indicator is generated from $Bernoulli(r)$ distribution with probability equals to r. If acceptance indicator equals to 1, the next state in Metropolis-Hastings iterations is updated $A_j^{*1} = A_j^*$. Otherwise $A_j^{*1} = A_j^t$.

(d) Iterative updates.
For Metropolis-Hastings iterations $h = 1, 2, ..., 99$, take A_j^{*h} as the current state, repeat the above steps to update the next state in Metropolis-Hastings sequences $A_j^{*1}, A_j^{*2}, ..., A_j^{*100}$. Finally, the next state of Gibbs iteration A_j^{t+1} is updated with the last state in Metropolis-Hastings iterations:

$$A_j^{t+1} = A_j^{*H} \ where\ H\ is\ the\ last\ state\ in\ MH\ iterations$$

7. Update mutation rate $\mathbf{M} = M_1, M_2, ..., M_J$.

(a) Conditional posterior distribution.

Parameters are updated one by one in the order $M_1^0, M_2^0, ..., M_J^0, M_1^1, M_2^1, ..., M_J^1$, ..., $M_1^t, M_2^t, ..., M_J^t, M_1^{t+1}, M_2^{t+1}, ..., M_J^{t+1}$ conditional on all other parameters are fixed. Within each Gibbs iteration, the Metropolis-Hastings algorithm proposes and updates the parameter for 100 times. Then the next Gibbs iteration is chosen from last 10 states of Metropolis-Hastings iterations.

$$\begin{aligned}&f(M_j^t|\mathbf{S}, b^t, G^t, \mathbf{H^t}, \mathbf{R^t}, \mathbf{A^t}, M_1^t, ..., M_{j-1}^t, M_{j+1}^t, ..., M_J^t)\\ &\propto f(\mathbf{n_j^t}|R_j^t, A_j^t, M_j^t) f(M_j^t|H_j^t)\end{aligned}$$

The marginal distribution of M_j^t can hardly be integrated. Thus the proportional posterior distribution is used and Metropolis-Hastings algorithm is applied.

(b) Proposal distribution.
Beta distribution is used as the proposal distribution to generate a candidate value M_j^* in the neighborhood based on the value of current state M_j^t. The transition probability and reverse transition probability are as follows:

$$q(M_j^*|M_j^t) = Beta(\alpha = \frac{1 + 1000M_j^t}{1 - M_j^t}, \beta = 1002)$$
$$q(M_j^t|M_j^*) = Beta(\alpha = \frac{1 + 1000M_j^*}{1 - M_j^*}, \beta = 1002)$$

(c) Acceptance probability.
Compute the acceptance ratio and determine the acceptance probability:

$$acceptance\ ratio = \frac{f(\mathbf{n_j^t}|R_j^t, A_j^t, M_j^*) f(M_j^*|H_j^t) \cdot q(M_j^t|M_j^*)}{f(\mathbf{n_j^t}|R_j^t, A_j^t, M_j^t) f(M_j^t|H_j^t) \cdot q(M_j^*|M_j^t)}$$

Then acceptance probability is determined as:

$$r = min\left\{1, \quad \frac{f(\mathbf{n_j^t}|R_j^t, A_j^t, M_j^*) f(M_j^*|H_j^t) \cdot q(M_j^t|M_j^*)}{f(\mathbf{n_j^t}|R_j^t, A_j^t, M_j^t) f(M_j^t|H_j^t) \cdot q(M_j^*|M_j^t)}\right\}$$

The acceptance indicator is generated from $Bernoulli(r)$ distribution with probability equals to r. If acceptance indicator equals to 1, the next state in Metropolis-Hastings iterations is updated $M_j^{*1} = M_j^*$. Otherwise $M_j^{*1} = M_j^t$.

(d) Iterative updates.
For Metropolis-Hastings iterations $h = 1, 2, ..., 99$, take M_j^{*h} as the current state, repeat above steps to update the next state in Metropolis-Hastings sequences $M_j^{*1}, M_j^{*2}, ..., M_j^{*100}$. Finally, the next state of Gibbs iteration M_j^{t+1} is updated

with the last state in Metropolis-Hastings iterations:

$$M_j^{t+1} = M_j^{*H} \textit{ where } H \textit{ is the last state in } MH \textit{ iterations}$$

8. Burn-in and thinning.
The Markov Chain will converge to the stationary distribution eventually after sufficient iterations. The effect of initial values can be ignored when iterations are large enough. In the simulation study, random initial values are assigned to different Markov chains and some starting points may over sample the regions that are rare events. The beginning samples are not stabilized to the stationary distribution and should be disregarded. In this simulation, the first 1600 iterations are removed from the final samples.

9. Convergence and diagnosis.
A major consideration in MCMC simulations is the convergence of Markov chains. The simulated chains are expected to fully explore the target distribution. Multiple chains initiated with different starting values are expected to ignore initial status and approximate the posterior distribution after long enough simulations. A common method to assess the MCMC convergence is to analyze and compare the differences between multiple chains.

The Gelman-Rubin statistic diagnose the MCMC convergence by analyzing the differences between multiple Markov chains. The convergence is evaluated by comparing the estimated between-chains and within-chains variances. Large differences between these variances indicate non-convergence.

Suppose there are M simulated chains with length T. Given a model parameter θ, let θ_{mt} be the value at the tth updates on the mth simulated chains, where $t = 1, 2, ..., T$ and $m = 1, 2, ..., M$. Let $\hat{\theta}_m$ and $\hat{\sigma}_m^2$ be the sample posterior mean and variance of the mth chain. The between-chains variance B and within-chains variance W are given by

$$\begin{aligned} B &= \frac{T}{M-1} \sum_{m=1}^{M} \left(\hat{\theta}_m - \hat{\theta}\right)^2 \\ W &= \frac{1}{M} \sum_{m=1}^{M} \hat{\sigma}_m^2 \end{aligned} \tag{S1}$$

The pooled variance $\widehat{V} = \frac{T-1}{T} W + \frac{M+1}{MT} B$ is an unbiased estimator of the marginal posterior variance of θ under certain stationarity conditions (Gelman and Rubin 1992). The potential scale reduction factor (PSRF) is defined to be the ratio of $\widehat{V}$ and W. If

the simulated M chains converge to the target distribution, then PSRF should be close to 1. The PSRF is given as below, where $\hat{d}$ is the degrees of freedom estimate of a t distribution.

$$R_c = \sqrt{\frac{\hat{d}+3}{\hat{d}+1}\frac{\hat{V}}{W}}$$

According to (Brooks and Gelman 1997) , if for all parameters the conditions $R_c < 1.2$ are satisfied, then it can be concluded that multiple Markov chains are converged. If PSRF reaches beyond this threshold, then simulated chains may not explore the full posterior distribution or longer simulations are needed.

2 Real Data Application

In addition, we also explored the differences between association status and variant effect size estimated by multi-marker Bayesian model and single-marker Bayesian model. In multiple-marker analysis, the segment association status is estimated by the posterior mean of MCMC simulation. In single-marker analysis, the segment association status is derived as the mean of estimated association status of individual variants. We compare the estimations of genes TACC2, CSMD1 and CDH13, which have been recognized to associate with breast cancer in multiple literature [1, 2, 3]. Figure 3-5 plot the relative risk distribution, segment association status and variant counts on each gene segment of the target gene. The plots show that the distributions of the estimated allelic relative risk are similar in both models. However, multi-marker Bayesian model is more sensitive to moderate variants by considering the joint effects of neighboring SNPs on a segment. Take the segment 6 and 7 in CSMD1 gene for example, given that the SNP counts are the same, the relative risk distribution has a small increase from segment 6 to segment 7. Correspondingly, the multi-marker model has a sharp jump on association status estimations from around 0.2 to 0.8, while single-marker model has a mild increase on association status estimations from 0.2 to 0.4. On the other hand, the number of variants also affects the aggregation impact. For example, the segments 44 and 46 of CSMD1 gene have 32 and 9 variants respectively. Both segments have moderate relative risk distributions, multi-marker model has association status 0.8 and 0.6 while single-marker model has both association status around 0.4.

References

[1] Nathalie Conte et al. "TACC1–chTOG–Aurora A protein complex in breast cancer." In: *Oncogene* 22.50 (Nov. 2003), pp. 8102–8116. DOI: 10.1038/sj.onc.1206972.

[2] Changqing Ma et al. "Characterization CSMD1 in a large set of primary lung, head and neck, breast and skin cancer tissues." In: *Cancer Biology & Therapy* 8.10 (May 2009), pp. 907–916. DOI: 10.4161/cbt.8.10.8132.

[3] K. O. Toyooka et al. "Loss of expression and aberrant methylation of the CDH13 (H-cadherin) gene in breast and lung carcinomas." In: *Cancer research* 61 (11 June 2001), pp. 4556–4560. ISSN: 0008-5472. ppublish.

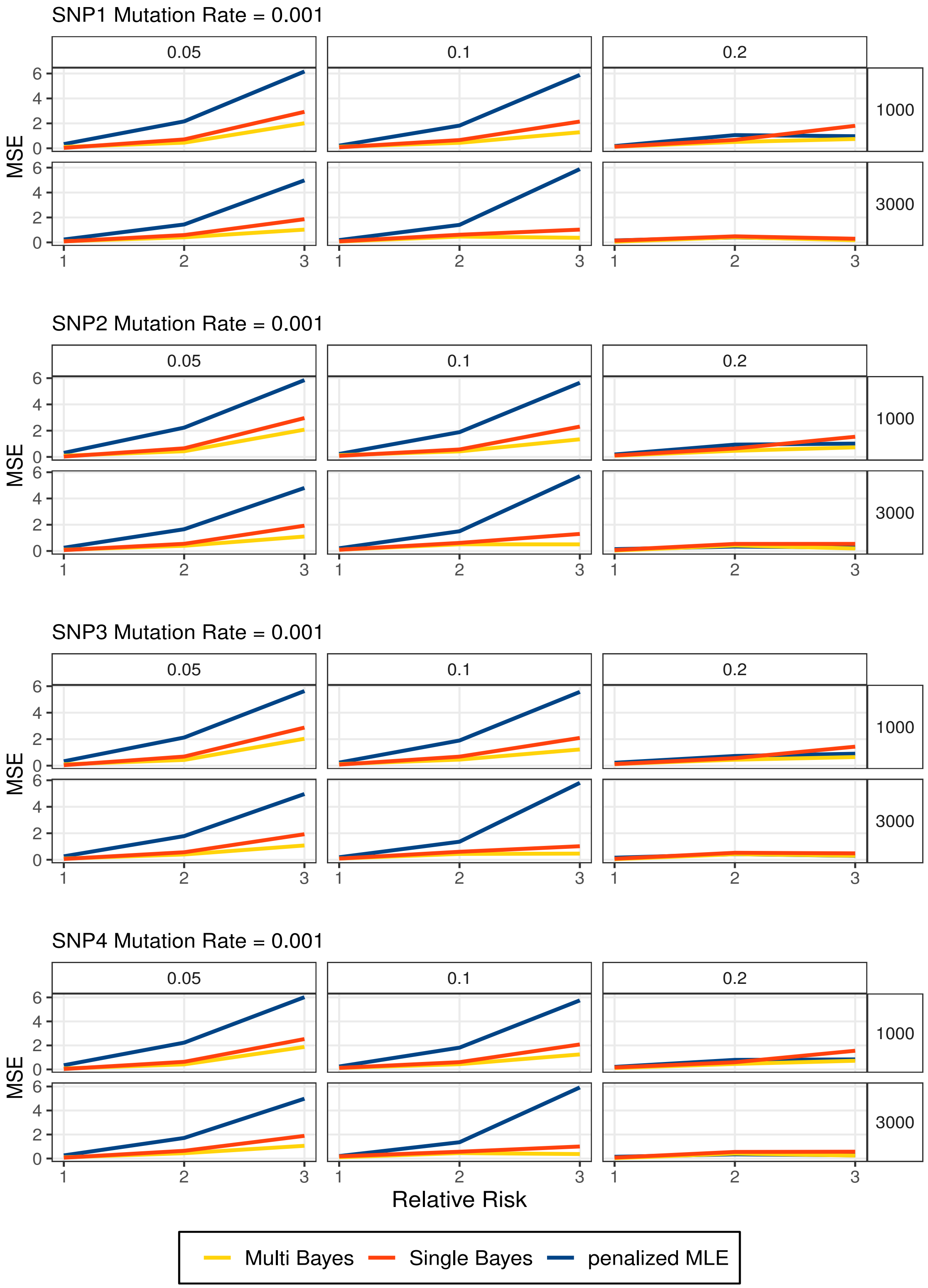

Figure S1: Mean square error of penalized MLE, Single-marker and Multiple-marker Bayesian models ($M = 0.001$).

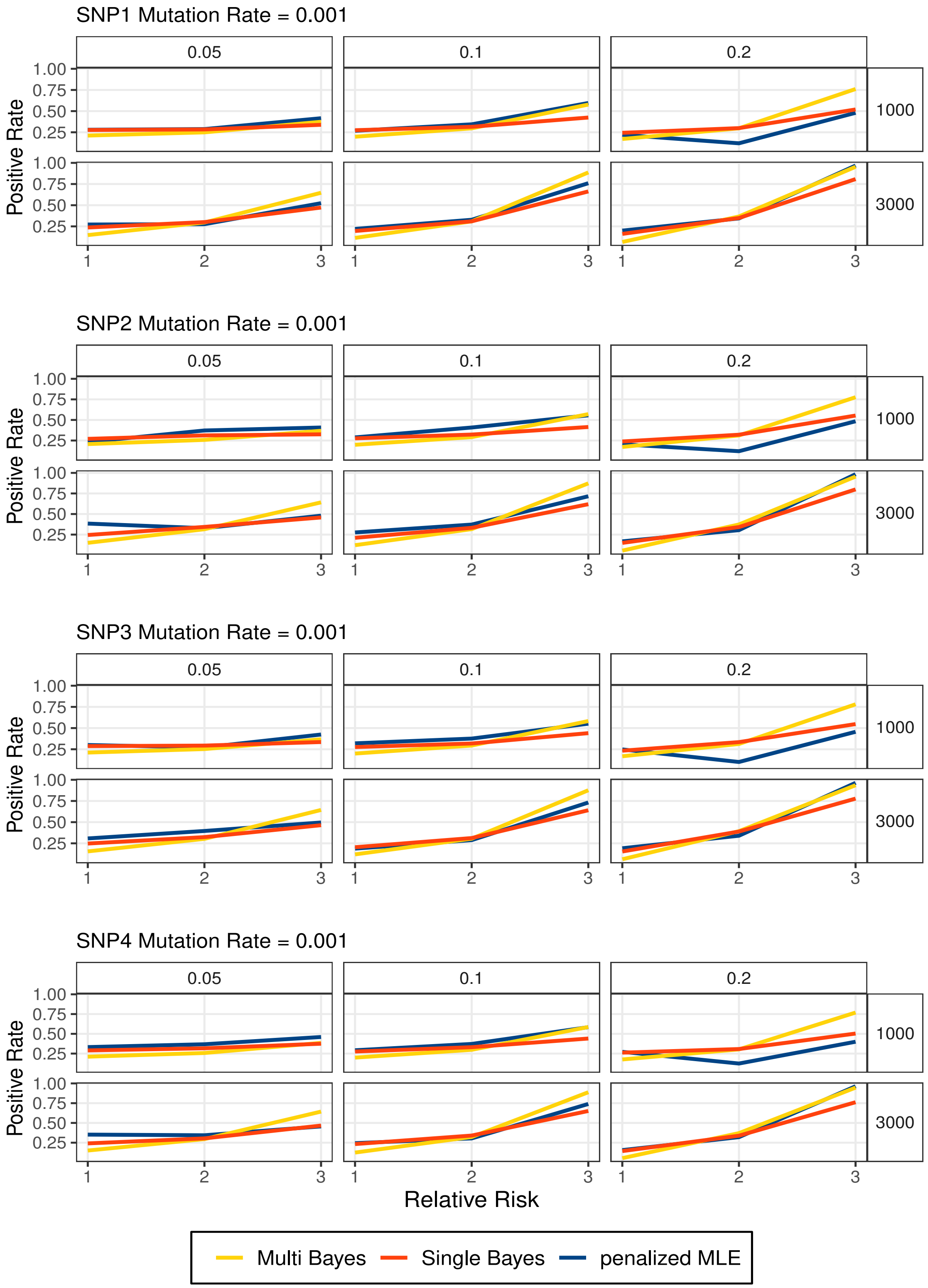

Figure S2: Estimated type I error rates ($R = 1$) and power ($R = 2, 3$) of penalized MLE, Single-marker and Multiple-marker Bayesian models ($M = 0.001$).

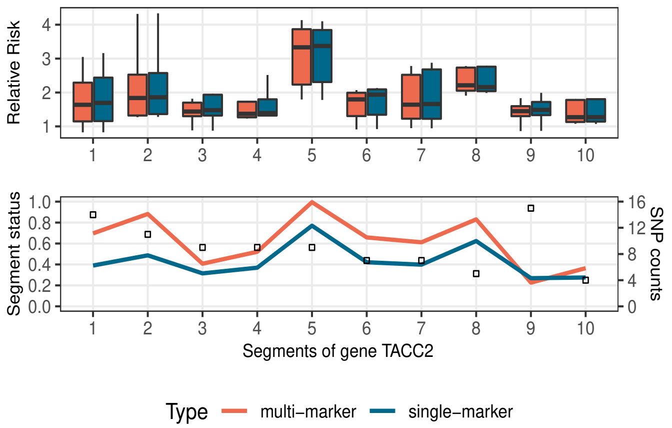

Figure S3: Estimated relative risk, segment status, and SNP counts of 10 segments on TACC2 gene

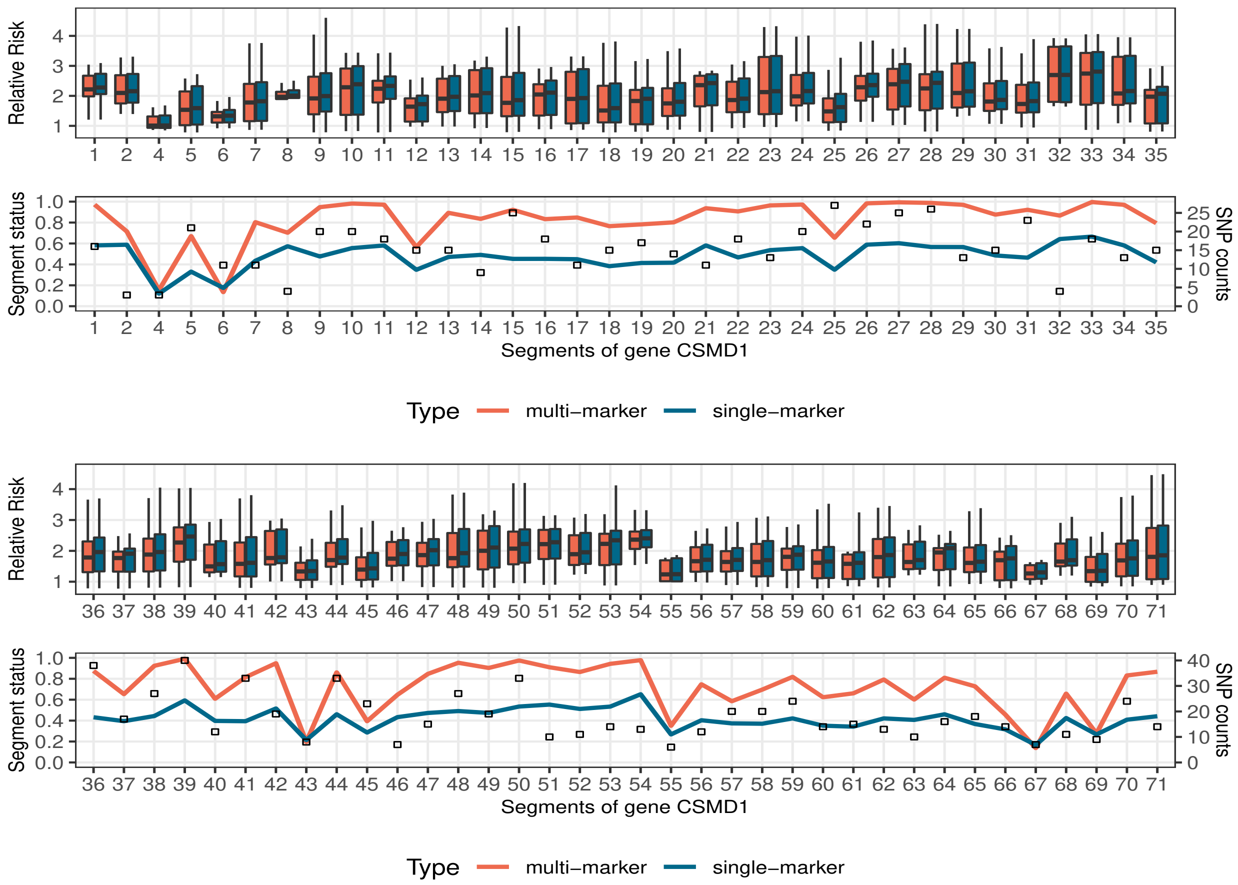

Figure S4: Estimated relative risk, segment status, and SNP counts of 71 segments on CSMD1 gene

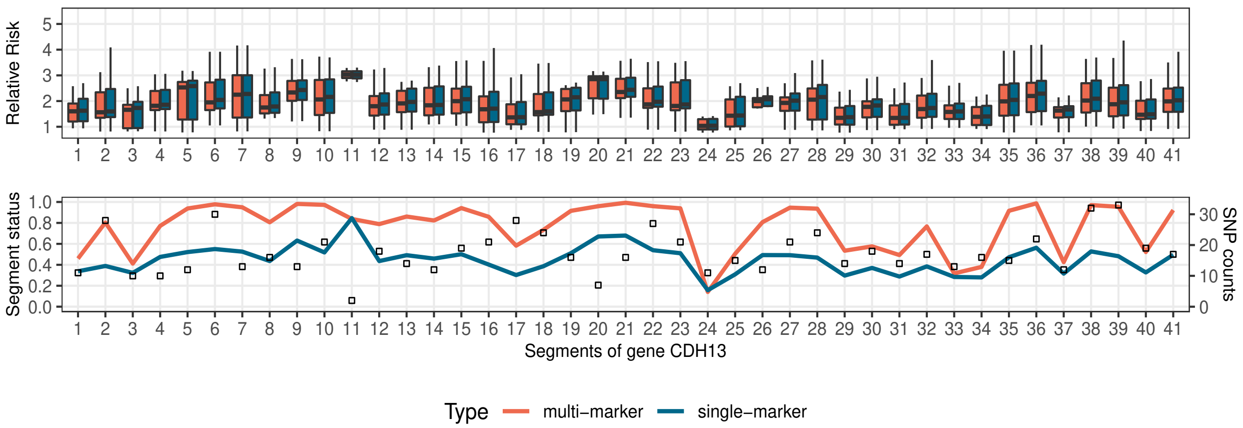

Figure S5: Estimated relative risk, segment status, and SNP counts of 41 segments on CDH13 gene
